# There Are No Differences in Positive Surgical Margin Rates or Biochemical Failure–Free Survival among Patients Receiving Open, Laparoscopic, or Robotic Radical Prostatectomy: A Nationwide Cohort Study from the National Cancer Database

**DOI:** 10.3390/cancers13010106

**Published:** 2020-12-31

**Authors:** Shyh-Chyi Chang, Ho-Min Chen, Szu-Yuan Wu

**Affiliations:** 1 Department of Urology, Lo-Hsu Medical Foundation, Lotung Poh-Ai Hospital, Yilan 265, Taiwan; mork2747@gmail.com; 2 Faculty of Medicine, National Yang-Ming University School of Medicine, Taipei 11221, Taiwan; 3 Department of Food Nutrition and Health Biotechnology, College of Medical and Health Science, Asia University, Taichung 413, Taiwan; homin.chen@gmail.com; 4 Big Data Center, Lo-Hsu Medical Foundation, Lotung Poh-Ai Hospital, Yilan 265, Taiwan; 5 Division of Radiation Oncology, Lo-Hsu Medical Foundation, Lotung Poh-Ai Hospital, Yilan, Taiwan; 6 Department of Healthcare Administration, College of Medical and Health Science, Asia University, Taichung 413, Taiwan; 7 Cancer Center, Lo-Hsu Medical Foundation, Lotung Poh-Ai Hospital, Yilan 265, Taiwan; 8 Graduate Institute of Business Administration, Fu Jen Catholic University, Taipei 242062, Taiwan; 9 School of Dentistry, College of Oral Medicine, Taipei Medical University, Taipei 110, Taiwan

**Keywords:** positive surgical margin, biochemical failure–free survival, robotic radical prostatectomy, open radical prostatectomy, laparoscopic radical prostatectomy

## Abstract

**Simple Summary:**

Few studies have evaluated oncologic outcomes in patients with prostate cancer (PC) receiving open, laparoscopic, or robotic radical prostatectomy (RP). To the best of our knowledge, this is the first and largest study to examine PSM and BFS rates in patients with PC undergoing open, laparoscopic, or robotic RP. After adjustment for confounders, no significant differences in PSM or BFS were noted among the patient groups.

**Abstract:**

*Purpose*: To estimate the rates of positive surgical margin (PSM) and biochemical failure–free survival (BFS) among patients with prostate cancer (PC) receiving open, laparoscopic, or robotic radical prostatectomy (RP). *Patients and Methods*: The patients were men enrolled in the Taiwan Cancer Registry diagnosed as having PC without distant metastasis who received RP. After adjustment for confounders, logistic regression was used to model the risk of PSM following RP. After adjustment for confounders, Cox proportional regression was used to model the time from the index (i.e., surgical) date to biochemical recurrence. *Results*: The adjusted odds ratios (95% CIs) of PSM risk after propensity score adjustment for laparoscopic versus open, robotic versus open, and robotic versus laparoscopic RP 95% CIs were 1.25 (0.88 to 1.77; *p* = 0.2064), 1.16 (0.88 to 1.53; *p* = 0.2847), and 0.93 (0.70 to 1.24; *p* = 0.6185), respectively. The corresponding adjusted hazard ratios (95% CIs) of risk of biochemical failure after propensity score adjustment were 1.16 (0.93 to 1.47; *p* = 0.1940), 1.10 (0.83 to 1.47; *p* = 0.5085), and 0.95 (0.74 to 1.21; *p* = 0.6582). *Conclusions*: No significant differences in PSM or BFS were observed among patients receiving open, laparoscopic, or robotic RP.

## 1. Introduction

With an estimated 1,100,000 new cases and 307,000 deaths in 2012, prostate cancer (PC) is the second most common cancer affecting men worldwide [1]. In Taiwan, PC is the fifth most common cancer in men. Treatment for PC, which depends on age, expected lifespan, and clinical staging, includes surgery, radiotherapy, hormone therapy, chemotherapy, active surveillance, or a combination of these [2]. According to the National Comprehensive Cancer Network [3], for men with newly diagnosed PC, the most important factors in initial treatment selection include the anatomic extent of disease (tumor [T] stage), histologic grade (Gleason score or grade group), serum prostate-specific antigen (PSA) concentration, and age, as well as risk stratification schema for localized PC. In patients with clinically localized PC, the recommended approaches for definitive therapy include radical prostatectomy (RP) or irradiation; the choice is largely a matter of patient preference [3], with most patients who choose surgery undergoing retropubic RP as the standard approach. Retropubic RP can be performed using either an open or minimally invasive approach (i.e., laparoscopic or robotic RP). Analyses of large databases indicate that robotic RP has increased rapidly in popularity, now constituting the modality used in the majority of cases [4,5].

A 2017 practice guideline jointly released by the American Urological Association, American Society for Radiation Oncology, and Society of Urologic Oncology (and endorsed by the American Society of Clinical Oncology in 2018) considers open and robotic RP to have comparable outcomes in continence recovery and sexual recovery based on previous studies [6,7,8,9,10,11,12]. Aside from the need for a smaller incision, the main advantages of laparoscopic or robotic RP mentioned in the guideline were perioperative outcomes such as reduced blood loss. Rates of positive surgical margin (PSM) and long-term biochemical failure–free survival (BFS) are less frequently addressed. Notably, the joint guideline was not based on evidence-based research or peer-reviewed articles but on specialist consensus. Whether robotic RP actually results in better outcomes in PSM or BFS in patients with PC of similar clinical stage (with risk stratification) remains unclear.

Few—if any—studies with sufficient sample size and follow-up duration have estimated the rates of PSM and BFS among patients with PC receiving open, laparoscopic, or robotic RP, especially in Asia. In the present study, the sample size was adequately large, the demographic and clinicopathological characteristics were balanced, and risk stratification was performed to estimate the rates of PSM and BFS among these specific patient groups.

## 2. Patients and Methods

### 2.1. Data Source

The study cohort was selected from the Taiwan Cancer Registry database (TCRD). We conducted a population-based cohort study using Taiwan National Health Insurance (NHI) Research Data (NHIRD) linked to the TCRD. The TCRD was established in 1979 and contains 97% of the cancer cases in Taiwan [13]. The NHIRD includes all medical claims data on disease diagnoses, procedures, drug prescriptions, demographics, and enrollment profiles of all beneficiaries [14]. The NHIRD and TCRD are linked by encrypted patient identifiers. NHIRD data are additionally linked to the Death Registry to ascertain the vital status and the cause of death of each patient. TCRD of Collaboration Center of Health Information Application contains detailed patient information, such as clinical stages, surgical procedures, techniques, radiotherapy, and chemotherapy (CT) regimens [15,16,17,18,19,20].

### 2.2. Study Cohort

The cohort was established from the Taiwan Cancer Registry. We enrolled patients who received a diagnosis of resectable PC and underwent RP between 1 January 2015, and 31 December 2015. The index date was the surgical date. The follow-up duration was the period from the index date to December 31, 2018. Our protocols were reviewed and approved by the Institutional Review Board of Tzu-Chi Medical Foundation (IRB109-015-B). Patient diagnoses were confirmed through the review of their pathological data, and patients who received a new diagnosis of resectable PC and underwent RP were confirmed to have no other cancer or distant metastasis. The type of RP we examined was surgery to remove the entire prostate gland and the surrounding lymph nodes as treatment for men with localized PC [21]. The inclusion criteria were a diagnosis of 5110 resectable PC with an indication for RP, being aged ≥20 years, and histologically confirmed cancer of the prostate in clinical stage T1–4 without distant metastasis (using the staging system of the American Joint Committee on Cancer). In our study, T1 means the cancer found during examination of the prostate. After tissue proof of prostate cancer by biopsy, patients with prostate cancer would chose RP, radiotherapy, or active surveillance depending on NCCN risk groups and expected patient’s survival time [3]. pT1 would be defined as the combined data of tissue proof or RP and recording to TCRD by the national professional cancer registry staffs. The exclusion criteria were a history of cancer before PC diagnosis (493 patients), unknown clinical or pathological stage (369 patients), unknown D’Amico risk classification (215 patients), unknown Gleason score (178 patients), unknown postoperative Gleason Grade group (99 patients), missing data on preoperative prostate-specific antigen (PSA) concentration (211 patients), clinically node-positive PC (428 patients), unclear margin status (110 patients), and nonadenocarcinoma histology (201 patients). To prevent potential interference with BFS, we excluded 1399 patients with PC who did not receive standard RP after PC diagnosis or who received additional treatment such as radiotherapy, androgen deprivation therapy, or chemotherapy after RP. However, if biochemical failure was confirmed, undergoing salvage radiation, androgen deprivation therapy, chemotherapy, and immune therapy did not disqualify patients from study inclusion. To compare their outcomes, the patients undergoing open, laparoscopic, and robotic RP were assigned to groups 1, 2, and 3, respectively.

Of the 1407 patients enrolled (Table 1), 315, 276, and 816 were in groups 1, 2, and 3, respectively. The mean follow-up duration after the index date was 36.67 months (standard deviation [SD] = 4.63 months). No significant between-group differences in any of the covariates, which included age, clinical T-stage, pathological T-stage, Gleason Grade group, Gleason score, preoperative PSA concentration, D’Amico risk classification, and hospital level (academic vs. nonacademic), were observed.

### 2.3. Endpoints

The endpoints were rates of PSM and BFS. For patients who have undergone RP, we defines biochemical failure as a serum PSA ≥0.2 ng/mL according to the definition of biochemical failure by American Urologic Association [22].

### 2.4. Statistical Analysis

#### 2.4.1. Demographic Characteristics

The patient characteristics were described according to the surgical modality. Normally distributed continuous data are presented as means and SDs, whereas nonnormally distributed continuous data are presented as medians and interquartile ranges. Categorical data are presented as numbers and proportions. Analysis of variance and the Kruskal–Wallis test were applied to parametric and nonparametric continuous data, respectively.

#### 2.4.2. Risk factors of PSM and Biochemical Failure

After adjustment for confounders, logistic regression was used to model the risk of PSM following RP by surgical modality. The odds ratios in the multivariate analysis were adjusted for age, clinical T-stage, pathological T-stage, Gleason Grade group, Gleason score, preoperative PSA concentration, D’Amico risk classification, and hospital level. After adjustment for confounders, the time from the index date to biochemical recurrence among the patients (by surgical modality) was modeled using Cox proportional regression. In the multivariate analysis, the hazard ratios (HRs) were adjusted for age, clinical T-stage, pathological T-stage, Gleason Grade group, Gleason score, preoperative PSA concentration, D’Amico risk classification, hospital level, and surgical margin status.

### 2.5. Adjusted Results

#### 2.5.1. Differences in Margin Positivity Based on Surgical Modality

In the logistic regression analysis of the effects of surgical modality and propensity score matching on margin positivity, the following covariates were adjusted for: age, clinical T-stage, pathological T-stage, Gleason Grade group, Gleason score, preoperative PSA concentration, D’Amico risk classification, and hospital level. Two methods were used to adjust the analysis: (1) classic adjustment, in which the relationships between the surgical modality and outcome were adjusted with the covariates through multivariate regression modeling, and (2) propensity score adjustment, in which conditional exchangeability was achieved using the propensity scores. Each propensity score was estimated for the probability that the patient would undergo RP using one of the three surgical modalities, given the covariates. The propensity scores were calculated using a multinomial logistic regression model; that is, the estimated regression coefficients of the multinomial regression model were multiplied with each patient’s characteristic value.

#### 2.5.2. Differences in BFS by Surgical Modality

Cox regression models for BFS were built using classic or propensity score adjustment. After adjustment for confounders, Cox proportional regression was used to model the time from the index date to biochemical failure among the patients. In the multivariate analysis, the HRs were adjusted for age, clinical T-stage, pathological T-stage, Gleason Grade group, Gleason score, preoperative PSA concentration, D’Amico risk classification, hospital level, and surgical margin status. Cox regression with propensity score adjustment was performed to evaluate the risk of biochemical failure associated with different treatment modalities. All analyses were performed using SAS version 9.3 of the SAS System for Unix (SAS Institute Inc., Cary, NC, USA). A two-tailed *p*-value of <0.05 was considered significant. BFS was assessed using the Kaplan–Meier method. Differences among treatment modalities were determined using the log-rank test.

## 3. Results

### 3.1. Clinicopathological Characteristics

Table 1 presents the demographic and clinicopathologic characteristics of the study population, stratified by surgical modality. No significant between-group differences were noted in the covariates of age, clinical T-stage, pathological T-stage, Gleason Grade group, Gleason score, preoperative PSA concentration, D’Amico risk classification, hospital level.

### 3.2. Risk of PSM and Biochemical Failure

The significant risk factors for PSM were advanced pathological T-stage (pT2-pT3b), high Gleason score (>6), Gleason Grade (>2), higher preoperative PSA concentration (>5 ng/mL), and undergoing surgery at a nonacademic hospital (Table 2). The significant risk factors for biochemical recurrence were advanced clinical T-stage (cT2-cT4), advanced pathological T-stage (pT2-pT3b), high Gleason score (>6), Gleason Grade (>2), higher preoperative PSA concentration (>5 ng/mL), intermediate-to-high D’Amico risk, undergoing surgery at a nonacademic hospital, and PSM (Table 2).

### 3.3. Associations of PSM with Surgical Modality

The unadjusted PSM rates were 44.1%, 49.6%, and 44.4% for open RP, laparoscopic RP, and robotic RP, respectively (Table 1). Table 3 presents the logistic regression comparisons of PSM rates among the three approaches. The unadjusted risk of PSM after robotic RP was not significantly lower than that after open RP (OR [95% CI] = 1.01 [0.78 to 1.31], *p* = 0.9430) or laparoscopic RP (OR [95% CI] = 0.81 [0.62 to 1.06], *p* = 0.1286). A significant difference was found in the risk of PSM between open and laparoscopic RP (OR [95% CI] = 1.25 [0.90 to 1.73]; *p* = 0.1806). After classic adjustment, the risk of PSM after laparoscopic RP or robotic RP was not significantly lower than that after open RP (adjusted OR [95% CI] = 1.33 [0.90 to 1.95]; *p* = 0.1530 and 1.19 [0.88 to 1.62]; *p* = 0.2626, respectively). No significant difference between laparoscopic and robotic RP was noted (adjusted OR [95% CI] = 0.90 [0.65 to 1.24], *p* = 0.5143). After propensity score adjustment, the adjusted ORs of PSM risk for laparoscopic versus open, robotic versus open, and robotic versus laparoscopic RP were 1.25 (0.88 to 1.77; *p* = 0.2064), 1.16 (0.88 to 1.53; *p* = 0.2847), and 0.93 (0.70 to 1.24; *p* = 0.6185), respectively.

### 3.4. Associations of BFS with Surgical Modality

The Cox proportional hazards model was used to analyze BFS (i.e., time to biochemical failure) by surgical modality (Table 4). Cox regression analysis was used to compare differences in biochemical failure by surgical modality. Table 4 presents the risk of biochemical failure among the three groups (unadjusted or using classic adjustment or propensity score adjustment). No significant between-group differences in BFS were noted regardless of whether the analysis involved unadjusted Cox regression or classic or propensity score adjustment (Table 4). The HRs of risk of biochemical failure after unadjusted Cox regression were 0.99 (0.75 to 1.30; *p* = 0.9503), 0.89 (0.71 to 1.11; *p* = 0.2924), and 0.90 (0.71 to 1.13; *p* = 0.3550) for laparoscopic versus open, robotic versus open, and robotic versus laparoscopic RP, respectively. After classic adjustment, the corresponding adjusted HRs of risk of biochemical failure were 1.16 (0.92 to 1.46; *p* = 0.2002), 1.04 (0.78 to 1.40; *p* = 0.7665), and 0.90 (0.70 to 1.15; *p* = 0.3979). After propensity score adjustment, the corresponding adjusted HRs of risk of biochemical failure were 1.16 (0.93 to 1.47; *p* = 0.1940), 1.10 (0.83 to 1.47; *p* = 0.5085), and 0.95 (0.74 to 1.21; *p* = 0.6582). For pT2 stages, which correspond to a about 50% of patients or even more, we have do the subgroup analysis for pT2 stages as Supplemental Table S2. There were similar outcomes of pT2 and overall stages (Table 3 and Table 4).

**Figure 1:**
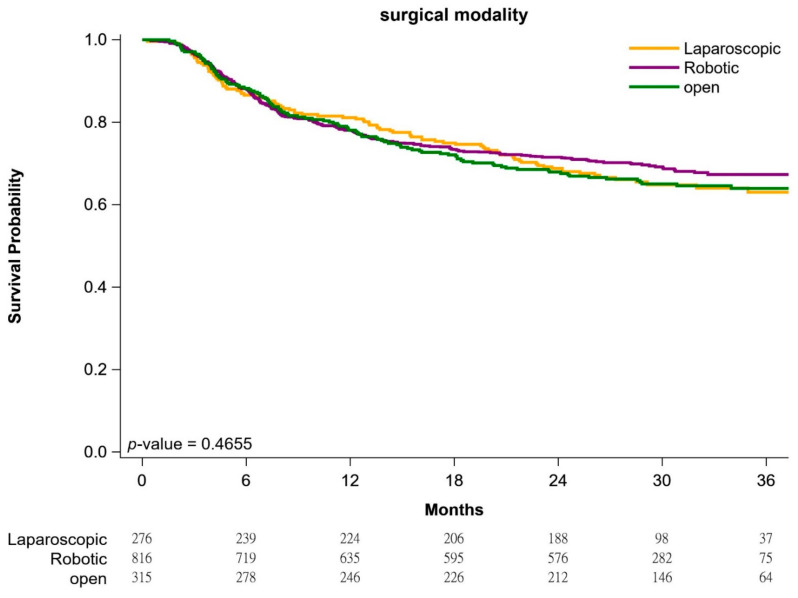
Biochemical failure–free survival curves, stratified by surgical modality.

Figure 1 presents the Kaplan–Meier BFS curves. The 3-year BFS rates for the patients receiving open, laparoscopic, and robotic RP were 69.7%, 68.9%, and 73.4%, respectively. As determined through the log-rank test, no significant between-group differences in BFS rate were observed (*p* = 0.4655).

## 4. Discussion

In 2014, a multinational, multi-institutional observational study (with a 15-year follow-up) was published by Sooriakumaran et al., comparing survival outcomes, including the PSM rate, among patients treated with radiotherapy or open, laparoscopic, or robotic RP [5]. Most patients were Caucasian and none were Asian [5], In other words, no studies comparing PSM following open, laparoscopic, or robotic RP in Asia have been conducted. Moreover, because non-Caucasian patients have higher PSM rates and narrower pelvic bony structure (mid-pelvic area) than do Caucasian patients [23,24,25], the outcomes from the study by Sooriakumaran et al. cannot be generalized to Asian patients. The PSM rates in the present study are higher than those reported by Sooriakumaran et al. and are consistent with those presented in other studies regarding the impact of ethnicity on RP surgical margins [23,24,25]. As presented in Table 1, the PSM rates were comparable among the groups: 44.1%, 49.6%, and 44.4% for open, laparoscopic, and robotic RP, respectively (*p* = 0.2783). The differences in results of the present study and that of Sooriakumaran et al. may be attributed to the fact that Asian patients have narrower pelvic bony structures, and also to the fact that less than 4% of patients with cT3-cT4 PC received RP in their study, whereas more than 22% did so in the present study (Table 1) [5]. The association of more advanced clinical T-stage with higher PSM rate in the present study appears to be in line with a finding from another study noting higher PSM rates among patients with PC and advanced T-stage [26]. The PSM rate for pT2 in our study were around 45% among the three surgical techniques and higher PSM rate than PSM rate for pT2 in German and Italian studies [27,28]. The reasons might be a different racial population, different pelvic bony structure, and different clinical stage distribution which could be the novelty and related with some different findings from other reports [5,23,24,25,26]. In addition, we also have done the subgroup analysis for pT2 stages as Supplemental Table S2, there were similar outcomes of pT2 and overall stages (Table 3 and Table 4). To the best of our knowledge, the present study is the first to assess outcomes of PSM and BFS among Asian patients with PC receiving open, laparoscopic, or robotic RP.

As presented in Table 1, the demographic and clinicopathological characteristics were comparable among the three groups. By contrast, they were significantly different among the corresponding groups in the study by Sooriakumaran et al. Furthermore, a clear bias in the selection of surgical modality (open, laparoscopic, or robotic RP) was present in that study [5]. Their patients in the robotic RP group were also considerably younger and had lower clinical and pathological stage, preoperative PSA concentration, and D’Amico risk [5]. Moreover, more than one third of the data on clinical stage and D’Amico risk classification in this group was missing [5]. Taken together, this indicates that the findings of Sooriakumaran et al. may not be completely accurate [5]. To the best of our knowledge, no randomized controlled trials (RCTs) have compared the rates of PSM and BFS among patients with PC undergoing open, laparoscopic, or robotic RP. Only small-scale RCTs have examined functional outcomes, complication rates and quality of life among patients with PC receiving open, laparoscopic, or robotic RP [29]. According to a meta-analysis of RCTs, some small-scale RCTs have indicated that patients undergoing minimally invasive and open RP have similar quality-of-life outcomes with regard to urinary and sexual recovery and function, as well as serious complication rates. However, none have assessed oncologic outcomes such as PSM or BFS [29]. Undergoing RP using minimally invasive surgical techniques was associated with shorter hospital stay and fewer blood transfusions performed [29]. However, high-quality data were not used in addressing oncologic outcomes. The present study is the first with a sufficient sample size to examine PSM and BFS rates (with no missing data) among patients with PC with homogeneous demographical and clinicopathological characteristics undergoing open, laparoscopic, or robotic RP (Table 1).

Table 2 presents the results of the multivariate analysis of PSM and biochemical failure in the study population. No significant associations of surgical modality with PSM and biochemical failure were noted (Table 2). The significant risk factors for PSM were advanced pathological T-stage (pT2-pT3b), high Gleason score (>6), Gleason Grade (>2), higher preoperative PSA concentration (>5 ng/mL), and undergoing surgery at a nonacademic hospital (Table 2). Risk factors including high preoperative PSA concentration, high Gleason score, high Gleason Grade, and advanced pathological T-stage in patients with PSM are in line with those identified in previous studies [30,31,32,33]. The association of undergoing surgery at a nonacademic hospital with high PSM risk may be attributed to the association of hospitals with low patient volume with PSM risk. This finding is consistent with that of Sooriakumaran et al. [5]. The significant risk factors for biochemical recurrence were advanced clinical T-stage (cT2-cT4), advanced pathological T-stage (pT2-pT3b), high Gleason score (>6), Gleason Grade (>2), higher preoperative PSA concentration (>5 ng/mL), higher D’Amico risk (intermediate to high), undergoing surgery at a nonacademic hospital, and PSM (Table 2). Risk factors such as higher D’Amico risk, higher preoperative PSA, high Gleason score, high Gleason Grade, advanced pathological T-stage, and advanced clinical T-stage of biochemical recurrence were consistent with those reported in previous studies [34,35,36,37]. Whether the covariates of age, clinical T-stage, pathological T-stage, Gleason score, Gleason Grade, preoperative PSA concentration, D’Amico risk classification, and hospital level were unadjusted or underwent classic or propensity score adjustment, no significant between-group differences in PSM rate were observed (Table 3). The outcomes demonstrate that receiving RP using minimally invasive surgical techniques (compared with other modalities) does not necessarily lead to superior oncologic outcomes (e.g., PSM).

As presented in Table 4, the follow-up duration was sufficiently long (mean 36 months). Moreover, the sample size was large. We did not examine overall survival among patients by surgical modality because the mortality rate was very low (approximately 1–2%; Table 1). By contrast, a retrospective study by Coughlin et al. in 2018 included only 326 patients with PC (whose data were extracted from another RCT), and the follow-up duration was shorter (24 months) [38]. In addition, the comparison performed by Coughlin et al. was restricted to that of BFS rates between patients with PC receiving open and robotic RP; laparoscopic RP was not evaluated [38]. In the present study, the crude biochemical recurrence rates were approximately 30–35% and did not differ significantly among the three groups (Table 1). Notably, the biochemical failure rate in our study was higher than that reported by Coughlin et al. This can be ascribed to the fact that the present study included more patients with cT3-cT4 PC and locally advanced D’Amico risk classification as well as higher PSM rate [38]. Thus, additional treatments such as radiotherapy, androgen deprivation therapy, or chemotherapy may be vital to Asian patients with PC receiving RP (whether open, laparoscopic, or robotic). If additional treatments were also categorized under biochemical failure by Coughlin et al. [38], their reported biochemical failure rate may be similar to ours, because no additional treatments were examined in the present study. According to our systemic review, this is the first study to examine BFS rates among patients with PC receiving open, laparoscopic, and robotic RP. We had a large sample size, and the demographic and clinicopathological characteristics of the study population were comparable among the three groups. Moreover, numerous covariates with impacts on BFS such as age, clinical or pathological stage, Gleason score, Gleason Grade, preoperative PSA concentration, D’Amico risk classification, hospital level, and PSM were adjusted as necessary (Table 2 and Table 4). By contrast, in the study by Coughlin et al., numerous confounding factors were not taken into account with regard to adjustment (including propensity score adjustment) for the multivariate analysis. The BFS rate did not significantly differ among the three groups (Figure 1).

The strengths of the present study are as follows. The sample size was large, the follow-up duration was sufficiently long, and the covariates used were consistent. The clinical characteristics of patients in the three groups were comparable. Most major covariates such as age, clinical stage, pathological stage, Gleason score, Gleason Grade, preoperative PSA concentration, D’Amico risk classification, hospital level, and PSM were taken into account in the adjusted analyses (Table 2, Table 3, Table 4). To the best of our knowledge, this is the first and largest study to examine PSM and BFS rates in patients with PC undergoing open, laparoscopic, or robotic RP. No significant differences were found in PSM or BFS rate with regard to the surgical modality used (Table 3, Table 4 and Figure 1). The present findings serve as a reference for clinical practice and prospective clinical trials.

This study has some limitations. First, in the multivariate analysis (Table 2), PSM and BFS were affected by hospital levels. It would be due to the technical level [39]. In the present study, the patient who were treated by the expert surgeons and novice, should be analyzed separately for the objectives. However, the expert surgeons and novice could not be distinguished and defined in our database. In fact, there is no well definition for expert surgeons and novice for open, laparoscopic, or robotic RP. Thus, we have separated the hospitals levels (academic or non-academic hospitals) for sensitivity analysis of multivariate analysis after propensity scores adjustment comparing PSM and BFS stratified by hospital levels as Supplemental Table S1. The trend of PSM and BFS were similar as Table 3 and Table 4 among the three surgical techniques in academic or non-academic hospitals. Second, the surgeon having more experiences of RP for more patients might be considered as an experienced surgeon, although different surgeons might vary the learning curves [9]. Nevertheless, the exact numbers of RP performed by the same surgeon considered as an experienced and well-trained surgeon have been unclear. Owing to the information security, the identifications of patients and surgeons were delinked in TCRD. Therefore, the specific surgeon could not be identified for the technical levels of each modalities. However, the hospital levels have been adjusted in the multivariate analysis to resolve the bias of the technical level of each modality in academic or non-academic hospitals (Table 3 and Table 4). Third, because all the patients were Asian in ethnicity, the corresponding ethnic susceptibility remains unclear. Fourth, there is no subgroups of focal or ono-focal PSM in TCRD. The association of biochemical failure and focal or ono-focal PSM might be bias. However, the PSM status were adjusted in the multivariate analysis of BCF, and there has been scarce studies to have the data of focal or non-focal PSM. We have tried our best to adjust all potential covariates for all available confounding factors. Fifth, the Taiwan Cancer Registry does not contain information regarding dietary habits, socioeconomic status, or body mass index, all of which may be risk factors for PSM or BFS. Therefore, extrapolation of the present findings to non-Asian populations should be done with caution. To obtain crucial information on population specificity and disease occurrence, large-scale randomized trials comparing carefully selected patients undergoing suitable treatments are warranted. However, considering the magnitude and significance of the observed effects in this study, these limitations likely did not affect the conclusions.

## 5. Conclusions

No significant differences in PSM or BFS were noted among patients with PC receiving open, laparoscopic, or robotic RP.

## Figures and Tables

**Table 1:** Demographic and Clinicopathological Characteristics, Stratified by Surgical Modality.

		Open RP *N* = 315		Laparoscopic RP *N* = 276		Robotic RP *N* = 816		
Characteristic		n	(%)	n	(%)	n	(%)	*p*-Value
Age	Mean (SD)	66.4	(6.8)	66.8	(6.4)	66.1	(6.7)	0.4661
	Median (IQR)	67	(62–71)	67	(62–72)	66	(62–71)	
	20–59	49	(15.6)	41	(14.9)	130	(15.9)	0.9004
	60–69	165	(52.4)	146	(52.9)	444	(54.4)	
	70+	101	(32.1)	89	(32.2)	242	(29.7)	
Clinical T-stage	cT1	84	(26.7)	75	(27.2)	195	(23.9)	0.2839
	cT2	149	(47.3)	133	(48.2)	436	(53.4)	
	cT3-cT4	82	(26.0)	68	(24.6)	185	(22.7)	
Pathological T-stage	pT1	96	(30.5)	83	(30.1)	237	(29.0)	0.1884
	pT2	152	(48.3)	137	(49.6)	432	(52.9)	
	pT3a	37	(11.7)	30	(10.9)	76	(9.3)	
	pT3b	30	(9.5)	26	(9.4)	71	(8.7)	
Gleason Grade group	1	3	(1.0)	2	(1.0)	9	(1.1)	0.2766
	2	40	(12.7)	35	(12.7)	133	(16.3)	
	3	162	(51.4)	142	(51.4)	434	(53.2)	
	4	47	(14.9)	43	(15.6)	103	(12.6)	
	5	63	(20.0)	54	(19.6)	137	(16.8)	
Gleason score	2–6	34	(10.8)	37	(13.4)	142	(17.4)	0.0951
	3 + 4	110	(34.9)	89	(32.2)	274	(33.6)	
	4 + 3	62	(19.7)	53	(19.2)	160	(19.6)	
	8–10	109	(34.6)	97	(35.1)	240	(29.4)	
Preoperative PSA (ng/mL)	Mean (SD)	15.8	(15.9)	17.6	(17.8)	15.8	(16.6)	0.3483
	Median (IQR)	10.3	(6.9–18.0)	10.4	(7.0–20.5)	10.3	(6.7–17.6)	
Preoperative PSA (ng/mL)	0–5	37	(11.7)	32	(11.6)	94	(11.5)	0.6540
	6–10	110	(34.9)	95	(34.4)	285	(34.9)	
	11–20	86	(27.3)	82	(29.7)	233	(28.6)	
	20+	82	(26.0)	67	(24.3)	204	(25.0)	
D’Amico risk classification	Localized—Low	13	(4.1)	15	(5.4)	58	(7.1)	0.1117
	Localized—Intermediate	93	(29.5)	69	(25.0)	219	(26.8)	
	Localized—High	122	(38.7)	120	(43.5)	338	(41.4)	
	Locally advanced	87	(27.6)	72	(26.1)	201	(24.6)	
Hospital level	Academic center	258	(81.9)	225	(81.5)	673	(82.5)	0.7251
	Non-academic center	57	(18.1)	51	(18.5)	143	(17.5)	
Follow-up duration (months)	Mean (SD)	36.1	(4.4)	37.2	(5.0)	36.2	(4.7)	
Surgical margin	Negative	176	(55.9)	139	(50.4)	454	(55.6)	0.2783
	Positive	139	(44.1)	137	(49.6)	362	(44.4)	
Biochemical failure		112	(35.6)	96	(34.8)	253	(31.0)	0.2502
Death		8	(2.5)	4	(1.4)	11	(1.3)	0.3534

IQR, interquartile range; SD, standard deviation; RP, radical prostatectomy; T, tumor; PSA, prostate-specific antigen.

**Table 2:** Multivariate Analysis of Positive Surgical Margin and Biochemical Failure.

		Positive Surgical Margin			Biochemical Failure		
Covariate		OR	(95% CI)	*p*-Value	HR	(95% CI)	*p*-Value
Surgical modality	Open RP	Ref		0.3095	Ref		0.6292
	Laparoscopic RP	1.22	(0.89–1.66)		0.98	(0.73–1.31)	
	Robotic RP	1.34	(0.91–1.97)		1.09	(0.86–1.37)	
Age	20–59	ref		0.9430	Ref		0.3779
	60–69	1.03	(0.73–1.47)		0.83	(0.63–1.09)	
	70+	0.99	(0.67–1.44)		0.83	(0.62–1.12)	
Clinical T-stage	cT1	Ref		0.2327	Ref		0.0001
	cT2	0.98	(0.50–1.76)		1.71	(1.06–3.11)	
	cT3-cT4	0.93	(0.49–1.65)		2.45	(1.33–5.11)	
Pathological T-stage	pT1	Ref		<0.0001	Ref		<0.0001
	pT2	3.38	(300–7.23)		1.32	(1.11–4.40)	
	pT3a	4.68	(2.57–8.51)		2.01	(1.33–3.04)	
	pT3b	5.15	(2.73–9.71)		3.21	(2.12–4.87)	
Gleason Grade group	1-2	Ref		0.0132	Ref		<0.0001
	3	1.51	(1.27–4.29)		1.32	(0.61–1.71)	
	4	1.66	(1.13–4.71)		1.82	(1.16–3.40)	
	5	2.99	(1.61–5.43)		2.69	(1.55–4.82)	
Gleason score	≥6	Ref		0.0432	Ref		<0.0001
	7	1.94	(1.15–3.29)		1.52	(0.89–2.58)	
	8	1.72	(1.03–3.16)		1.70	(1.06–3.00)	
	9+	2.28	(1.26–4.14)		2.44	(1.41–4.23)	
Preoperative PSA (ng/mL)	0–5	Ref		<0.0001	Ref		0.0019
	6–10	1.25	(0.79–1.99)		0.69	(0.45–1.06)	
	10–20	1.88	(1.17–3.02)		0.95	(0.63–1.45)	
	20+	3.31	(1.97–5.55)		1.23	(0.80–1.88)	
D’Amico risk classification	Localized—low	Ref		0.3438	Ref		0.0001
	Localized—intermediate	0.87	(0.40–1.86)		3.71	(1.05–13.07)	
	Localized—high	0.83	(0.39–1.76)		4.34	(1.25–15.09)	
	Locally advanced	0.54	(0.22–1.30)		8.03	(2.23–28.83)	
Hospital level	Academic center	Ref		0.0474	Ref		0.0002
	Non-academic center	1.07	(1.05–1.10)		1.58	(1.24–2.01)	
Surgical margin	Negative	-			Ref		0.0005
	Positive				1.47	(1.18–1.82)	

RP, radical prostatectomy; CI, confidence interval; T, tumor; PSA, prostate-specific antigen; OR, odds ratio; HR, hazard ratio; Ref, reference group.

**Table 3:** Logistic Regression Analysis of Positive Surgical Margin by Surgical Modality.

	Laparoscopic *v* Open, OR (95% CI)	*p*-Value	Robotic *v* Open, OR (95% CI)	*p*-Value	Robotic *v* Laparoscopic, OR (95% CI)	*p*-Value
Positive surgical margin						
Unadjusted logistic regression	1.25 (0.90–1.73)	0.1806	1.01 (0.78–1.31)	0.9430	0.81 (0.62–1.06)	0.1286
Logistic regression with classic adjustment (with covariates mentioned in Table 1 *)	1.33 (0.90–1.95)	0.1530	1.19 (0.88–1.62)	0.2626	0.90 (0.65–1.24)	0.5143
Logistic regression with propensity score adjustment (matched with covariates mentioned in Table 1 *)	1.25 (0.88–1.77)	0.2064	1.16 (0.88–1.53)	0.2847	0.93 (0.70–1.24)	0.6185

CI, confidence interval; OR, odds ratio * Covariates mentioned in Table 1: age, clinical T-stage, pathological T-stage, postoperative Gleason score, Gleason Grade group, preoperative prostate-specific antigen concentration, D’Amico risk classification, and hospital level.

**Table 4:** Cox Regression Analysis of Biochemical Failure Rates by Surgical Modality.

	Laparoscopic *v* Open, HR (95% CI)	*p*-Value	Robotic *v* Open, HR (95% CI)	*p*-Value	Robotic *v* Laparoscopic, HR (95% CI)	*p*-Value
Biochemical failure rates						
Unadjusted Cox regression	0.99 (0.75–1.30)	0.9503	0.89 (0.71–1.11)	0.2924	0.90 (0.71–1.13)	0.3550
Cox regression classic adjustment (with covariates mentioned in Table 1 *)	1.16 (0.92–1.46)	0.2002	1.04 (0.78–1.40)	0.7665	0.90 (0.70–1.15)	0.3979
Cox regression with propensity scores for adjustment (matched with covariates mentioned in Table 1 *)	1.16 (0.93–1.47)	0.1940	1.10 (0.83–1.47)	0.5085	0.95 (0.74–1.21)	0.6582

CI, confidence interval; HR, hazard ratio * Covariates mentioned in Table 1: age, clinical T-stage, pathological T-stage, Gleason Grade group, postoperative Gleason score, preoperative prostate-specific antigen concentration, D’Amico risk classification, hospital level, and surgical margin status.

## Data Availability

Restrictions apply to the availability of these data. Data was obtained from Taiwan Ministry of Health and Welfare and are available from Szu-Yuan Wu with the permission of Institutional Review Board of Tzu-Chi Medical Foundation (IRB109-015-B).

## Supplementary Materials

The following are available online at https://www.mdpi.com/2072-6694/13/1/106/s1, Table S1: Sensitivity analysis of multivariate analysis after propensity scores adjustment comparing Positive surgical margin and biochemical failure rates stratified by hospital levels, Table S2: Multivariate analysis after propensity scores adjustment comparing Positive surgical margin and biochemical failure rates in pathologic T2 stages.

## Availability of Data and Material

We used data from the National Health Insurance Research Database (NHIRD) and Taiwan Cancer Registry database (TCRD). The authors confirm that, for approved reasons, some access restrictions apply to the data underlying the findings. The data utilized in this study cannot be made available in the manuscript, the supplemental files, or in a public repository due to the “Personal Information Protection Act” executed by Taiwan’s government, starting from 2012. Requests for data can be sent as a formal proposal to obtain approval from the ethics review committee of the appropriate governmental department in Taiwan. Specifically, links regarding contact info for which data requests may be sent to are as follows: http://nhird.nhri.org.tw/en/Data_Subsets.html#S3 and http://nhis.nhri.org.tw/point.html.

## Abbreviations

RP	Radical prostatectomy
PC	Prostate cancer
BFS	Biochemical failure–free survival
PSM	Positive surgical margin
CI	Confidence interval
T	Tumor
PSA	Prostate-specific antigen
AJCC	American Joint Committee on Cancer
SD	Standard deviation
OR	Odds ratio
HR	Hazard ratio
RCT	Randomized controlled trial
