# Supplementary material for: There Are No Differences in Positive Surgical Margin Rates or Biochemical Failure–Free Survival among Patients Receiving Open, Laparoscopic, or Robotic Radical Prostatectomy: A Nationwide Cohort Study from the National Cancer Database
Source: Cancers (Basel). 2020 Dec 31;13(1):106. doi: 10.3390/cancers13010106 (PMC7795659; doi:10.3390/cancers13010106)
Supplement: Supplementary file 1 [file cancers-13-00106-s001.pdf]

Table S1. Sensitivity analysis of multivariate analysis after propensity scores adjustment comparing Positive surgical margin and biochemical failure rates stratified by hospital levels.

**Oncologic Outcomes**	**Laparoscopic *v* open, OR (95% CI)**	***P* - value**	**Robotic *v* open, OR (95% CI)**	***P*-value**	**Robotic *v* laparoscopic, OR (95% CI)**	***P* - value**
**Positive surgical margin**						
**Academic centers**						
Logistic regression with propensity score adjustment (matched with covariates mentioned in Table 1*)	1.16 (0.76–1.89)	0.3156	1.25 (0.79–1.80)	0.3906	0.97 (0.69–1.30)	0.7074
**Non-Academic centers**						
Logistic regression with propensity score adjustment (matched with covariates mentioned in Table 1*)	1.31 (0.77–2.86)	0.4175	1.20 (0.77–2.75)	0.4736	0.96 (0.68–2.10)	0.8074
	Laparoscopic *v* open, HR (95% CI)	*P*-value	Robotic *v* open, HR (95% CI)	P Value	Robotic *v* laparoscopic, HR (95% CI)	*P*-value
**Biochemical failure rates**						
**Academic centers**						
Cox regression with propensity scores for adjustment (matched with covariates mentioned in Table 1**)	1.06 (0.97–1.50)	0.2879	1.20 (0.74–1.39)	0.5194	0.97 (0.63–1.19)	0.5991
**Non-Academic centers**						
Cox regression with propensity scores for adjustment (matched with covariates mentioned in Table 1**)	1.31 (0.72–2.16)	0.4762	1.20 (0.61–2.67)	0.6177	0.99 (0.60–2.90	0.8212

CI, confidence interval; OR, odds ratio; HR, hazard ratio. *Covariates mentioned in Table 1: age, clinical T-stage, pathological T-stage, postoperative Grade group, postoperative Gleason score, preoperative prostate-specific antigen concentration, and D'Amico risk classification. **Covariates mentioned in Table 1: age, clinical T-stage, pathological T-stage, postoperative Grade group, postoperative Gleason score, preoperative prostate-specific antigen concentration, D'Amico risk classification, and surgical margin status.

Table S2. Multivariate analysis after propensity scores adjustment comparing Positive surgical margin and biochemical failure rates in pathologic T2 stages

**Oncologic Outcomes**	**Laparoscopic *v* open, OR (95% CI)**	***P*-value**	**Robotic *v* open, OR (95% CI)**	***P*-value**	**Robotic *v* laparoscopic, OR (95% CI)**	***P*-value**
**Positive surgical margin**						
Logistic regression with propensity score adjustment (matched with covariates mentioned in Table 1*)	1.29 (0.61–2.19)	0.2293	1.15 (0.61–1.88)	0.4955	0.91 (0.71–1.98)	0.7991
	Laparoscopic *v* open, HR (95% CI)	*P*-value	Robotic *v* open, HR (95% CI)	P Value	Robotic *v* laparoscopic, HR (95% CI)	*P*-value
**Biochemical failure rates**						
Cox regression with propensity scores for adjustment (matched with covariates mentioned in Table 1**)	1.88 (0.81–1.91)	0.5110	1.17 (0.81–2.10)	0.5251	0.71 (0.59–1.71)	0.7001

CI, confidence interval; OR, odds ratio; HR, hazard ratio. *Covariates mentioned in Table 1: age, clinical T-stage, pathological T-stage, postoperative Grade group, postoperative Gleason score, preoperative prostate-specific antigen concentration, and D'Amico risk classification. **Covariates mentioned in Table 1: age, clinical T-stage, pathological T-stage, postoperative Grade group, postoperative Gleason score, preoperative prostate-specific antigen concentration, D'Amico risk classification, and surgical margin status.
